# Risk Factors Associated With Frequent Acute Exacerbations of Asthma

**DOI:** 10.7759/cureus.11090

**Published:** 2020-10-22

**Authors:** Sheeba F Ansari, Mubeen Memon, Ratan Kumar, Amber Rizwan

**Affiliations:** 1 Internal Medicine, Liaquat University of Medical and Health Sciences, Jamshoro, PAK; 2 Pulmonology, Liaquat University of Medical and Health Sciences, Jamshoro, PAK; 3 Pulmonology, Civil Hospital, Khairpur, PAK; 4 Family Medicine, Jinnah Postgraduate Medical Centre, Karachi, PAK

**Keywords:** asthma, acute exacerbations, risk factors

## Abstract

Introduction: Asthma can lead to fatigue, frequent hospital visits, psychological problems, and learning problems in children. One of the complications of asthma is its life-threatening acute exacerbation. It is important to identify precipitating factors responsible for frequent acute exacerbations of asthma.

Methods: This case-control study was conducted in the pulmonology ward of Liaquat University of Medical and Health Sciences, Jamshoro, from May 2019 to February 2020. Sampling was done by convenient probability technique. The case group was identified as patients with two or more episodes of acute exacerbation of asthma and the control group was identified as asthmatic patients without acute exacerbation in the last year.

Results: Factors leading to acute exacerbation of asthma include number of asthma attacks in the past seven days (4.9 ± 3.4 vs. 2.2 ± 2.0; p < 0.0001) and number of nights with troublesome cough in the past 28 days (12.2 ± 8.1 vs. 4.3 ± 3.1; p < 0.0001). Participants with recent upper respiratory tract infection (38.4% vs. 10%; odds ratio [OR] 5.62), smoking history (30.7% vs. 12%; OR 3.25), gastroesophageal reflux disease (26.9% vs. 8.0%; OR 4.2) and non-adherence to medication (26.9% vs. 8.0%; OR 4.2) were more likely to experience from exacerbation of asthma.

Conclusion: It is important to identify risk factors that may cause acute exacerbation of asthma in the patients. Patients should be educated of the risk factors and complications of the exacerbation episode of asthma.

## Introduction

Asthma is a clinical condition affecting all age groups. The World Health Organization (WHO) defines asthma as “a disease characterized by breathlessness and wheezing which vary in severity and frequency from person to person” [1]. It occurs due to chronic reversible inflammation of the air passages and airway hyperactivity causing swelling of airways, and hence decreased airflow out of the lungs. According to the Centers for Disease Control and Prevention (CDC), 10% of the world's population suffers from asthma, 5% of which is classified as severe asthma [2].

Asthma has been associated with various complications including fatigue, frequent hospital visits, psychological problems, and learning problems in children. One of the complications of asthma is its life-threatening acute exacerbation, characterized by increased symptoms, lung function deterioration, and increased airway inflammation [3]. The global literature has identified various risk factors that might be responsible for acute exacerbation of asthma [3-5]. Exacerbations are frequently associated with upper respiratory tract infection (URTI) [3]. Other risk factors identified, in a study by Chaudhry et al., are female gender, illiteracy, patient unawareness, allergen and drug exposure, medication non-compliance, anxiety, and blood eosinophilia [4]. One-fifth of asthmatic patients have episodes of acute exacerbation of asthma [5].

It's very important to diagnose asthma exacerbation early in its course and manage accordingly. Despite being a common health issue in Pakistan, there is very limited data available on asthma in a local setting, particularly related to risk factors associated with acute exacerbation of asthma. In this study, we aim to determine the risk factors associated with acute exacerbation in the asthmatic population of Pakistan.

## Materials and methods

This case-control study was conducted in the pulmonology ward of Liaquat University of Medical and Health Sciences, Jamshoro, from May 2019 to February 2020. Sampling was done by convenient probability technique. Frequent exacerbations were defined as two or more in the previous year. Fifty-two patients with frequent acute exacerbations of asthma from the emergency unit were included in the case group. Exacerbation was identified using the diagnostic criteria defined by Global Initiative for Asthma (GINA) [6]. Fifty stable asthmatic patients, with no history of exacerbation in the last year, were enrolled from the outpatient department as the control group. Patients in the age group of 18-70 years belonging to either gender were included in the study. Patients with severe lung diseases such as parenchymal diseases, tuberculosis, chronic obstructive pulmonary disease, and other illnesses such as chronic kidney disease and congestive heart failure were excluded from the study.

A self-administrated questionnaire was used to note participants’ age, gender, number of asthma attacks in the last seven days, number of nights with troublesome cough in the past 28 days, and incidence of URTI in the past 28 days. The numerical values, such as age, number of attacks in the past week, and number of nights with troublesome cough in the last 28 days were presented as means and standard deviations. Categorical data such as gender, smoking, exercise, family history, and gastroesophageal reflux disease (GERD) were presented as frequencies and percentages. Chi-square test, t-test and odds ratio (OR) formula were used as appropriate. A p-value less than 0.05 indicated that there was a difference between variables of case and control and null hypothesis was not valid.

## Results

The overall mean age of participants was 35 ± 15 years. Number of asthma attacks in the past seven days (4.9 ± 3.4 vs. 2.2 ± 2.0; p < 0.0001), number of nights with troublesome cough in the past 28 days (12.2 ± 8.1 vs. 4.3 ± 3.1; p < 0.0001), URTI (38.4% vs. 10%; OR 5.62), smoking (30.7% vs. 12%; OR 3.25), gastrointestinal reflux disease (26.9% vs. 8.0%; OR 4.2), and non-adherence to medication (26.9% vs. 8.0%; OR 4.2) were found to be associated with frequent exacerbations in asthmatic patients (Table 1).

**Table 1:** Comparison of variables between case and control groups NSAID, non-steroidal anti-inflammatory drug; OR, odds ratio. *Significant result, **non-significant result. ^a^Value was calculated using chi-square. ^b^Independent t-test was applied. ^c^Value was calculated using the OR formula.

Variables	Case (N=52)	Control (N=50)	p-value/OR
Age (mean ± SD)	34 ± 14	39 ± 16	0.09^a,^*
Gender			0.3^a,^*
Male	21 (40.38%)	25 (50%)	
Female	31 (59.6%)	25 (50%)	
Number of attacks in the past 7 days (mean ± SD)	4.9 ± 3.4	2.2 ± 2.0	<0.0001^b,^**
Number of nights with troublesome cough in the past 28 days (mean ± SD)	12.2 ± 8.1	4.3 ± 3.1	<0.0001^b,^**
Upper respiratory tract infection in the past 28 days	20 (38.4%)	05 (10%)	5.62^c^
Smoking	16 (30.7%)	06 (12%)	3.25^c^
Exercise	11 (21.1%)	12 (24%)	0.84^c^
NSAIDs	04 (7.6%)	04 (8.0%)	0.95^c^
Beta blockers	02 (3.8%)	03 (6.0%)	0.62^c^
Gastrointestinal reflux disease	14 (26.9%)	04 (8.0%)	4.2^c^
Family history of asthma	08 (15.3%)	03 (6.0%)	2.8^c^
Non-adherence to medication	14 (26.9%)	04 (8.0%)	4.2^c^

## Discussion

In this study, acute exacerbation of asthma was more frequent in patients who reported higher number of asthma attacks in the last seven days and higher number of nights with troublesome cough in the past 28 days. URTIs in the past 28 days, smoking, gastroesophageal reflux, and non-adherence to medication were also associated with significantly increased risk of exacerbation. The number of patients with a family history of asthma was higher in case group compared to control; however, the difference was not significant. Few other local studies have shown similar results. They found female gender, URTI, medication non-compliance, allergen, and drug exposure as potential risk factors for frequent exacerbations in asthmatic patients [4].

This study adds to the limited local data available related to risk factors responsible for the increased frequency of exacerbations of asthma. However, the study has its limitations. First, it was a single institution study; hence, the diversity in sample might be reduced. Second, since patients were asked retrospectively about the number of asthma attacks in the past seven days or number of nights with troublesome cough in the past 28 days, there might a possibility of memory bias. Another limitation was that the outcomes of treating modifiable risk factors were not studied in this study.

The pathogenesis of airflow obstruction in asthma exacerbations occurs because of concentric smooth muscle contraction, edema of airway wall, and obstruction of lumen of airway with mucus [7,8]. There are various factors identified in the literature that may cause acute asthma exacerbations. These include viruses, allergens including dust and pollen, occupation exposure such as grain, wood or metal, variation in hormones, exercise, stress, and various drugs such as non‐steroidal anti‐inflammatory drugs and β‐blockers [9].

In this study, URTI was associated with increased acute exacerbation of asthma. URTI may result in the production of various pro‐inflammatory mediators, including interleukin-1, interleukin-6, and interleukin-8 [9]. These pro-inflammatory markers attract inflammatory cells such as neutrophils, lymphocytes, and eosinophils. Patients presenting with acute exacerbation of asthma due to respiratory tract infections have significantly higher percentages of neutrophils in their sputum [10]. Neutrophils are responsible for destroying airway epithelial cells, and trigger an inflammatory reaction [11].

In this study, smoking was also identified as a risk factor for the increased frequency of acute exacerbation of asthma. In a prospective study from 2007, smoking was identified as the most powerful independent modifiable risk factor for frequent exacerbations of asthma [12]. Cigarette smoking, like respiratory infection, may result in increased neutrophils in airways [13].

In this study, GERD was identified as a risk factor. There are various possible explanations for the increased frequency of exacerbation of asthma in patients with concomitant GERD. GERD may directly induce asthma by an aspiration-induced response. It may also indirectly induce asthma through neurogenically induced inflammation [14].

Asthma is a highly prevalent global health problem with significant morbidity and mortality. It is important that physicians identify the risk factors responsible for exacerbations of asthma. Efforts should be made to increase patients’ awareness regarding disease and medication compliance, as a strategy to reduce the frequency of exacerbations. Interventions aimed at correcting the modifiable risk factors should be a part of management plan.

## Conclusions

Risk factors found to be associated with frequent exacerbations were a higher number of asthma attacks in the last seven days and a higher number of nights with troublesome cough in the past 28 days. Asthmatic patients who have had recent URTIs in the past 28 days, smokers, those who have gastrointestinal reflux, and those non-adherent to medication are at a higher risk of having an asthmatic exacerbation. Further large-scale, multi-center studies are needed to assess the association of various variables and frequency of exacerbation of asthma.
